# Supplementary material for: Loancorhynchus catrillancai gen. et sp. nov., a new swordfish (Xiphioidei, Blochiidae) from the Middle Eocene of central Chile
Source: PeerJ. 2019 Mar 26;7:e6671. doi: 10.7717/peerj.6671 (PMC6440463; doi:10.7717/peerj.6671)
Supplement: Data S1 — (I) Clarification of SGO.PV.6634 stratigraphic provenance; (II) List of Characters; (III) Phylogenetic analysis [file peerj-07-6671-s001.pdf]

SUPPLEMENTARY DATA

I) Clarification of SGO.PV.6634 stratigraphic provenance

The studied specimen SGO.PV.6634 was part of a large sample of fossils (mostly marine invertebrates) originally collected by Humberto Fuenzalida in 1935, as it is accounted in his field notes available at the Museo Nacional de Historia Natural (Santiago, Chile). The collection includes material from three different localities (Constitución, Punta Parrón and Faro Carranza) and three stratigraphic units. The provenance of each specimen was provided by San Martín (1946). Particularly, the latter author stated the following (see image below):

"*a big piece of sandstone presents numerous tubes which correspond to* Teredo*; but this material cannot be identified with any of the species neither with the* Teredo *sp., described in the assemblage from Constitución, thus, I think it is prudent to avoid any affirmation by now with respect to them. Furthermore, it should be considered that the available material* ...(from Faro Carranza)... *only offers tubes of Teredo.*"

Fam. TEREDINIDAE. Leach

Gén. Teredo. Linné 1757.

TEREDO sp.

Un gran trozo de areniscas presenta numerosos tubos correspondientes a Teredo; pero, este material no se puede i-dentificar con ninguna de las especies ni tampoco con el Tere-do sp, descrito en el conjunto proveniente de Constitución y, me parece prudente no afirmar nada por ahora respecto de ellos. Además, habría que tener en cuenta que el material a disposi-ción ofrece sólo tubos de Teredo.-

During 2008, during the revision of old collections at the MNHN, Sergio Soto Acuña (Universidad de Chile) found the mentioned sandstones with putative "teredo" remains. These remained together with other material originally labeled as wood remains by Humberto Fuenzalida (original label is still available). These "wood remains" were not studied neither mentioned by San Martín (1946). The lithology of these blocks was the same of the "Teredo" sandstone. Indeed, this block was prepared by the author of this research during 2008 and 2009, fiding that the "tubes" were indeed bones that belonged to fin rays of a bony fish. After that, the "wood remains" were also prepared, revealing the skull remains here studied. Both materials (the "teredo" and the "wood remains") were collected by H. Fuenzalida at Faro Carranza in 1935 (contra San Martín, 1946 who stated 1936 as the date of collection). This date is reflected in the labels of all the specimens from

Faro Carranza, and also in the field notes of H. Fuenzalida. Likely, the material from Constitución dates back to 1936, or else, it was a typo in San Martín (1946) Prior to preparation, the former “wood remains” were mostly obscured by sediment, while the “teredo” remains were visible in cross as tubes in longitudinal view and others in cross-section. These were indeed fin rays scattered within the block. After preparation, the fin rays and the skull elements were found to be consistent in size, preservation (3D), lithology of the hosting block, locality of provenance (Faro Carranza), date (1935) and collector (H. Fuenzalida), supporting that these belong to a single specimen.

II) List of characters and character states used for phylogenetic analysis. Modified from Fierstine and Monsch (2002).

1) lacrimal size relative to orbit diameter. 0) smaller; 1) larger.
2) Supratemporal groove. 0) present; 1) absent.
3) Pineal swelling. 0) absent; 1) present.
4) Snout length relative to body length. 0) 0.10-0.20; 1) 0.20-0.32; 0.32; **2) 0.49-0.56. (new state, following Nakamura, 1995).**
5) Rostrum. 0) absent; 1) present.
6) Depth:width ratio throughout most rostral length. 0) ≥1; 1) between 0.5 and 1.0; 2) ≤ 0.5.
7) Longitudinal ridges on rostrum. 0) absent; 1) present.
8) Nutrient canals. 0) two; 1) two or more.
9) Central canal. 0) absent; 1) present.
10) Tooth size. 0) relatively large (clearly discernable); 1) minute denticles.
11) Teeth, denticles or their alveoli. 0) two single rows; 1) multiple rows, may be separated in two bands; 2) single broad band of villiform teeth **(new state, after Fierstine, 1999).**
12) Length of lower jaw relative to body length. 0) 0.12-0.18: 1) 0.2-0.27.
13) Length of pectoral fin relative to body length. 0) 0.02-0.08; 1) 0.086-2.72.
14) Precaudal vertebrae. 0) 18-34; 1) 11-16.
15) Caudal vertebrae. 0) 10-12; **1) 13-14; 2) 15-16;** 3) 30-40. **(1 and 2, new states following Nakamura, 1995**).
16) Total vertebrae. 0) 62-64; 1) 26; 2) 24; 3) 40.
17) Neural spines present throughout most of vertebral column. 0) yes. 1) no.
18) Neural and haemal spines. 0) spiniform; 1) modified into small plate-like struture; 2) large, plate-like structure.
19) Vertebrae in caudal complex. 0) three; 1) five; 2) two.
20) Epurals. 0) three; 1) two; 2) one.
21) Parhypural. 0) autogenous; 1) fused to hypural plate.
22) Caudal rays. 0) 39-43; 1) 25-34.
23) Dorsal fin depressible into groove. 0) no; 1) yes.
24) Length of anal-fin base of all elements (spines, rays, finlets) relative to body length. 0) 0.15-0.28; 1) 0.32-0.40.
25) Pectoral fin-rays. 0) 23-25; 1) 18-19; 2) 8-17.
26) Denticles present in dorsal surface of the rostrum (Fierstine, 1999): 0, absent; 1, present. New Character.
27) villiform teeth: 0) absent; 1) present in rostrum; 2) present in rostrum and dentaries. New Character.
28) rostrum / jaw ratio: 0, equally larger or almost similar; 1) rostrum much larger. New Character.

III) Phylogenetic analysis

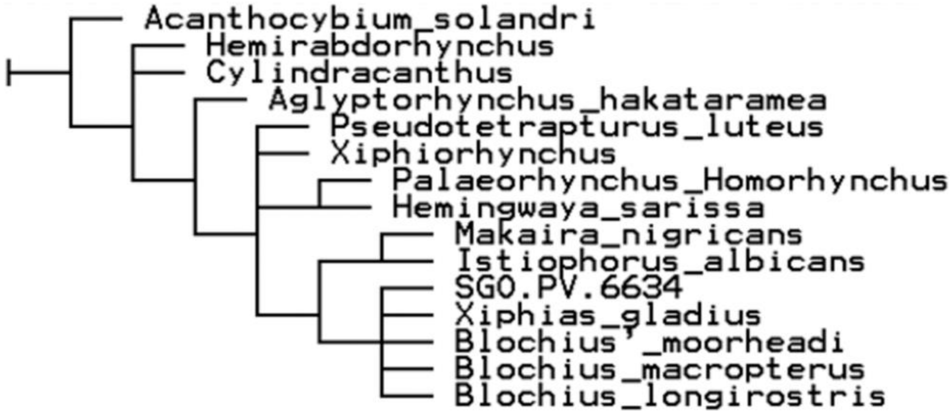

Figure 1: Strict consensus cladogram (68 steps; CI=0.735; RI=0.660) of 9 most parsimonious cladograms (MPC's), recovered with the datamatrix of Fierstine and Monsch (2002) modified here. Analysis considered Traditional Search, Wagner Trees (1,000 replicates; 1,000 trees to save per replication). *Acanthocybium solandri* is selected as outgroup.

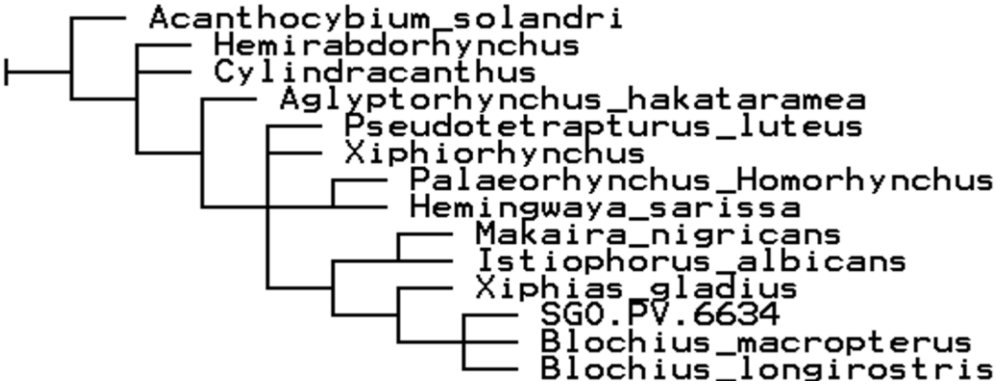

Figure 2: Strict consensus cladogram (65 steps; CI=0.754; RI=0.686) of 3 MPC's recovered after pruning the unstable taxon '*Blochius*' *moorheadi*. Analysyis considered Traditional Search (1,000 replicates; 1,000 trees to save per replication). Unstable taxa ('*Blochius*' *moorheadi* and *Xiphiorhynchus*) were detected with IterPcr script (Pol and Escapa, 2009). *Acanthocybium solandri* is selected as outgroup.

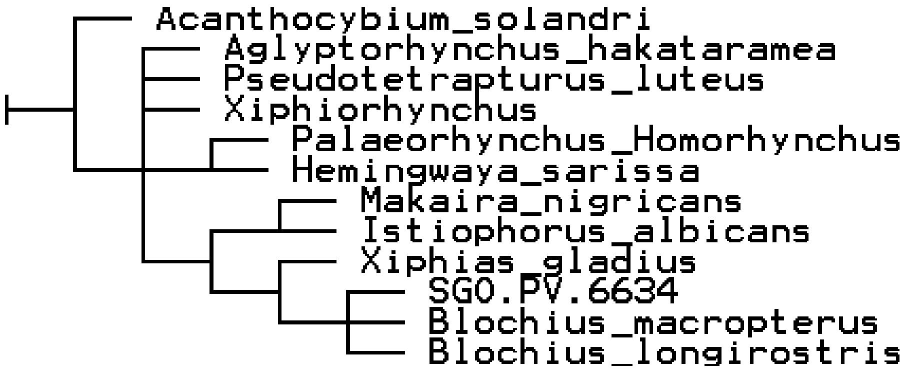

Figure 3: Strict consensus cladogram (64 steps; CI=0.766; RI=0.706) of 3 MPC's recovered after pruning the unstable taxon '*Blochius*' *moorheadi*, plus *Cylindracanthus* (considering it as an Acipenseriformes, following Parris and Grandstaff, 2001) and *Hemirhabdorhynchus*. Analysis considered Traditional Search (1,000 replicates; 1,000 trees to save per replication).

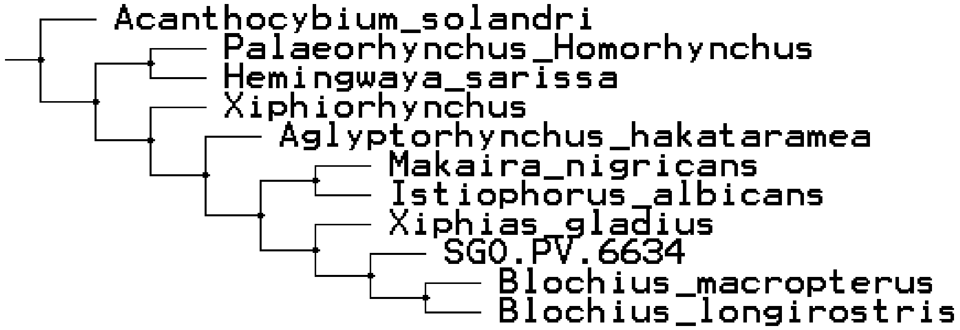

Figure 4: Single MPC (CI=0.750; RI=0.600), Implied Weighting (K-=3); New Technology Search; Ratchet. Pruned taxa: '*Blochius*' *moorheadi*, *Cylindracanthus*, *Hemirhabdorhynchus* and *Pseudotetrapturus luteus.*

ITERPCR: RESULTS.TXT FILE

The following reduced consensus is obtained after prunning the most unstable taxa:
==
==

Note: for consensus calculation, trees will be
temporarily collapsed (when min. branch length = 0)

Strict consensus of 9 trees

```
  ÚÄÄ Acanthocybium_solandri
  ³  ÚÄÄ Hemirabdorhynchus
ÃÄÄ´  ÃÄÄ Cylindracanthus
  ÀÄÄ´  ÚÄÄ Aglyptorhynchus_hakataramea
     ³  ³    ÚÄÄ Pseudotetrapturus_luteus
     ÀÄÄ´    ³       ÚÄÄ Palaeorhynchus_Homorhynchus
        ÀÄÄ>a´    ÚÄÄÁÄÄ Hemingwaya_sarissa
            ³    ³    ÚÄÄ Makaira_nigricans
            ÀÄÄ>a´  ÚÄÄÁÄÄ Istiophorus_albicans
                ÀÄÄ´    ÚÄÄ Xiphias_gladius
                   ÀÄÄ>b´    ÚÄÄ SGO.PV.6634
                        ÀÄÄ>bÅÄÄ Blochius_macropterus
                             ÀÄÄ Blochius_longirostris
```

Legends:
a: Xiphiorhynchus (5)
b: Blochius'_moorheadi (10)

The following taxa are unstable and collapse nodes in the strict consensus:

Xiphiorhynchus
Scoring the following characters may help to resolve its position:
    0 11 12 18 21 23 24

Blochius'_moorheadi
Scoring the following characters may help to resolve its position:
    0 1 3 5 6 11 12 16 19 20 24
==
===============
EVALUATING CHARACTERS INVOLVED IN ALTERNATIVE POSITIONS OF UNSTABLE TAXA TOOK 2 SECONDS
==
===============
